# In Silico Identification of Possible Inhibitors for Protein Kinase B (PknB) of *Mycobacterium tuberculosis*

**DOI:** 10.3390/molecules26206162

**Published:** 2021-10-12

**Authors:** Tatiana F. Vieira, Fábio G. Martins, Joel P. Moreira, Tiago Barbosa, Sérgio F. Sousa

**Affiliations:** 1 Associate Laboratory i4HB—Institute for Health and Bioeconomy, Faculdade de Medicina, Universidade do Porto, 4200-319 Porto, Portugal; tatianafvieira@gmail.com (T.F.V.); fabmaru@gmail.com (F.G.M.); work.joe1299@gmail.com (J.P.M.); 10180532@ess.ipp.pt (T.B.); 2 UCIBIO—Applied Molecular Biosciences Unit, BioSIM-Departamento de Biomedicina, Faculdade de Medicina, Universidade do Porto, 4200-319 Porto, Portugal

**Keywords:** *Mycobacterium tuberculosis*, serine/threonine protein kinases, PknB, virtual screening, molecular docking, molecular dynamics simulations, MM/GBSA

## Abstract

With tuberculosis still being one of leading causes of death in the world and the emergence of drug-resistant strains of *Mycobacterium tuberculosis* (Mtb), researchers have been seeking to find further therapeutic strategies or more specific molecular targets. PknB is one of the 11 Ser/Thr protein kinases of Mtb and is responsible for phosphorylation-mediated signaling, mainly involved in cell wall synthesis, cell division and metabolism. With the amount of structural information available and the great interest in protein kinases, PknB has become an attractive target for drug development. This work describes the optimization and application of an in silico computational protocol to find new PknB inhibitors. This multi-level computational approach combines protein–ligand docking, structure-based virtual screening, molecular dynamics simulations and free energy calculations. The optimized protocol was applied to screen a large dataset containing 129,650 molecules, obtained from the ZINC/FDA-Approved database, Mu.Ta.Lig Virtual Chemotheca and Chimiothèque Nationale. It was observed that the most promising compounds selected occupy the adenine-binding pocket in PknB, and the main interacting residues are Leu17, Val26, Tyr94 and Met155. Only one of the compounds was able to move the active site residues into an open conformation. It was also observed that the P-loop and magnesium position loops change according to the characteristics of the ligand. This protocol led to the identification of six compounds for further experimental testing while also providing additional structural information for the design of more specific and more effective derivatives.

## 1. Introduction

*Mycobacterium tuberculosis* (Mtb) is the pathogen responsible for the development of tuberculosis (TB). TB is, to this day, one of the most common causes of mortality, by a single pathogen, around the world. According to the World Health Organization (WHO), in 2019, Mtb was responsible for 10 million new infections globally, with an estimated 1.2 million deaths [1]. The treatment for TB is rather extensive and involves a combination of different antibiotics. If not performed correctly, it may lead to the development of drug-resistant strains such as the multi-drug-resistant and extensively drug-resistant strains (MDR and XDR-TB), which in turn, may cause higher transmissibility rates and larger outbreaks [2,3]. Finding additional or new strategies to control infection is of the utmost importance [4].

PknB is one of the 11 “eukaryotic-like” Serine/Threonine protein kinase (STPK) responsible for phosphorylation-mediated signaling in Mtb. It is a transmembrane protein with four extracellular PASTA (Penicillin binding proteins and Serine Threonine kinases-Associated) motifs and an intracellular catalytic kinase domain [5]. PknB is involved in many key bacterial processes such as cell wall synthesis, cell division and metabolism. Several studies have shown that its expression affects cell morphology and survival, with a depletion of PknB leading to the loss of bacterial viability [6,7,8]. In addition, it is essential for the reactivation of the cells from a hypoxic state, being an important “switch” between dormancy and resuscitation [9,10]. These aspects not only reinforce the significance of the role of PknB in Mtb, but also highlight its importance as a potential drug target.

**Figure 1:**
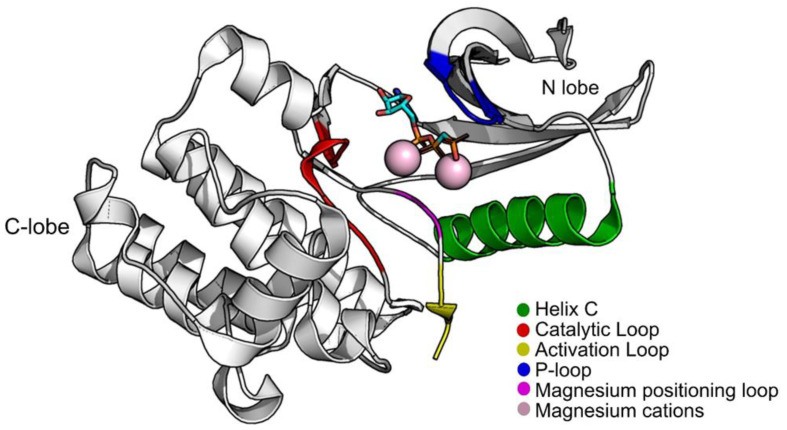
Structure of PknB (PDB:1O6Y) with groups that are important for its activity highlighted. Figure was produced using Pymol.

Structurally, PknB is divided into two lobes, an N-lobe and a C-terminal lobe. Between them, is the ATP pocket. As with most STPKs, it has five main groups that are indispensable for the catalytic process: helix C (residues 51–65); the P-loop (the phosphate-binding or glycine-rich loop) where the amides of the glycines coordinate the phosphates of ATP, functioning as a clamp (residues 18–23); the magnesium positioning loop (residues 156–158); the catalytic loop (residues 135–143); and the activation loop (residues 164–177) (Figure 1). PknB can be found in two conformations, open and closed, depending on the position of the helix C and the P-loop region. The open conformation corresponds to an inactive state of PknB, whereas the closed conformation corresponds to the active state. To be in a closed conformation and in an active state, a glutamic acid from the helix C must be close contact with a lysine near the P-loop, forming a salt bridge. This lysine is a fundamental piece for the catalysis as it orients the position of the phosphates of ATP [11,12].

Mycobacterial STPK only presents 30% sequence identity to eukaryotic STPK; however, the ATP-binding and catalytic mechanism are highly conserved [13]. There is a great interest in finding kinase inhibitors, mainly in cancer research, where it is important in signal regulation. In fact, the first crystal structure of PknB bound to a non-ATP analog was the result of an in silico screening of 40,000 compounds that concluded that mitoxantrone, a compound used in cancer treatment, could inhibit bacterial growth in culture. Since then, other inhibitors were found but showed limited activity on mycobacteria mainly due to poor permeability through the cell envelope [13,14]. Mycobacteria has a unique, thick lipid-rich envelope composed three layers: the capsule, the cell wall and the cell membrane. This highly hydrophobic cell envelope acts as a protective armor and hinders the diffusion of many compounds, including more hydrophobic molecules such as antibiotics [3,15,16]. Moreover, to reach the target, the drug must be able to cross the host cell membranes, as Mtb can be present in multiple intra- and extracellular entities, such as macrophages and necrotic granulomas [17].

**Figure 2:**
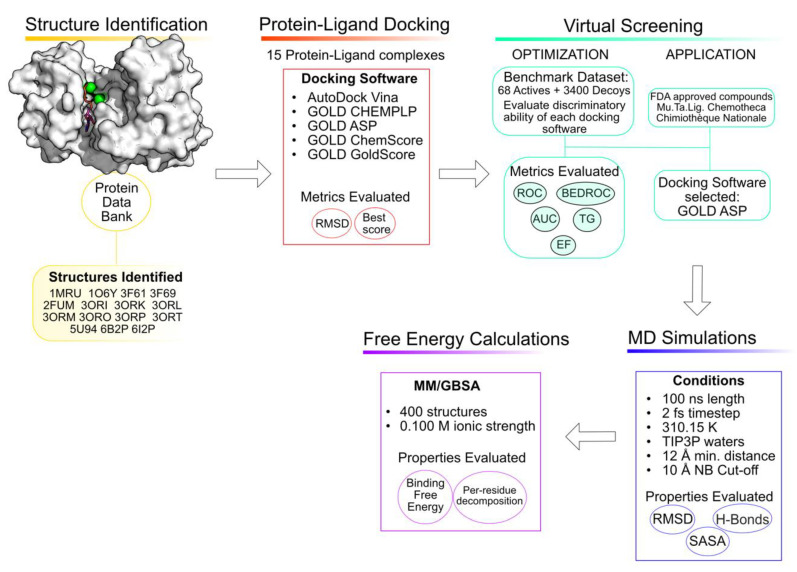
Workflow of the approach used in the current study.

Computer-aided drug discovery has been gaining more popularity due to the gains in computational power, the development of new programs and the exponential raise in the number of 3D protein targets available and deposited in the Protein Data Bank [18,19,20]. The usage of these computational tools in the early stages of drug design minimizes failures in later stages and makes the entire process more cost-efficient [21,22,23]. There are multiple published works in which the usage of these tools has led to the discovery of multiple promising compounds against PknB [24,25,26,27,28]. Consequently, this work aims to use computational tools to understand the binding pocket of PknB and to identify new promising inhibitors. To accomplish this goal, a combination of multiple computational methods was employed, including virtual screening, molecular dynamics simulations and free energy calculations [29]. An overview of the workflow is depicted in Figure 2.

## 2. Materials and Methods

### 2.1. Structure Identification

Structural information regarding the serine/threonine protein kinase (PknB) from *Mycobacterium tuberculosis* was obtained from the Protein Data Bank [30]. The X-ray structures and corresponding information of the targets selected are detailed in Table 1. A total of 15 protein structures and 8 ligands were used in the protein–ligand docking protocol validation stage. Only the catalytic domain of each structure (i.e., chain A) was used in this study.

Most of the molecular targets studied present magnesium (Mg^2+^) or manganese ions (Mn^2+^) in the active site. It has been demonstrated that magnesium ions have a stabilizing effect on ATP in order to allow for the kinase phosphorylation process to occur, even though its precise role is complex and not fully understood [36,37]. The mutations in the structures 3F61, 3F69, 3ORI, 3ORK, 3ORL, 3ORM, 3ORO, 3ORP and 3ORT were evaluated to further understand the role of specific residues in the dimerization and kinase regulation [31,32]. The manganese ions were typically added in such studies as substitutes for Mg^2+^.

Nearly all of the selected structures correspond only to the catalytic domain of PknB bound to an ATP analog. As mentioned previously, 2FUM was the first crystallized structure of PknB in complex with an inhibitor, a non-ATP analog, called mitoxantrone [18]. The structures containing antagonists (2FUM, 5U94 and 6B2P) are in the closed conformation, with Lys40 and Glu59 facing each other, except 3F69, in which the Glu59 is facing the outside of the pocket. The main different in these four structures, compared to the ones containing ATP analogs, is a small shift of the P-loop in the direction of the C-terminal lobe. All the antagonist molecules are in the nucleotide binding gap of ATP. The most important residues for the interactions with the antagonist molecules seem to be Leu17, Gly18, Val25, Ala38, Met 92, Glu93, Tyr94 and Val95 in the N-terminal lobe, and Met145 and Met155 in the C-terminal lobe [13,26,33].

### 2.2. Protein–Ligand Docking Protocol Validation

To evaluate and optimize the docking protocol and to select the best structure to represent this target in the virtual screening campaign, re-docking and cross-docking studies were performed. The goal of the re-docking consists in providing an assessment of the quality of the docking protocol in reproducing the pose of the crystallographic ligands in their specific targets [38]. In fact, the accuracy of different docking methods/scoring algorithms can vary significantly with the characteristics of the protein targets [38,39]. Several aspects of the protocol, such as the size and coordinates of the binding area, were then optimized toward minimizing the RMSD between the re-docked and crystallographic poses. Cross-docking consists in docking each ligand into all of the studied structures. It is an important method to evaluate the ability of each structure to correctly accommodate ligands from other structures and is a measure of its general usefulness [24]. Different scoring functions evaluate the target and the ligands in different ways [38,39,40,41]. Additionally, the type of target and characteristics of the ligand molecules can cause variation in the virtual screening (VS) results; therefore, it is important to consider as many scoring function (SF) alternatives as possible [39]. For this reason, five of the most widely used scoring functions in docking were used in this study: AutoDock Vina [42] and GOLD [43] CHEMPLP, ASP, ChemScore and GoldScore. The RMSD calculations were performed using DockRMSD [44]. The best performing SFs alongside the best protein structure, in terms of the RMSD (Å) comparison and docking score, were the ones selected to move to the next stage.

### 2.3. Virtual Screening Protocol Optimization

At this stage, the molecular targets that did not contain magnesium were removed, as these ions are an important part of the catalytic site, and the re-docking and cross-docking scores were decreased in the absence of these cations, confirming their importance for proper ligand placement. Similarly, the structures that contained manganese ions were also removed from the data set because its addition was only to evaluate their role in the stability of the protein [32] and their score in the re-docking and cross docking was lower when compared with the structures containing Mg^2+^ cations (Supplementary Materials—Table S1). From the five structures containing Mg^2+^ ions, only the two that presented the best results in the re-docking and cross-docking studies moved on to this stage.

To evaluate the quality of the VS protocol, the ability to discriminate between real binders and non-binders was evaluated by applying an active vs. decoys protocol. Actives are compounds that possess known experimental activity against PknB. Decoys are compounds randomly generated from the active molecule to have similar 1-d physico-chemical properties but different 2-d topologies to make them likely non-binders [37]. The ideal scoring function would rank the real binders better than decoys, but due to imperfections in the performance of the actual SF, that is not always the case [23]. Hence, the chosen SF to move on to the screening of large databases of compounds is the one that provides the best results in terms of early recognition metrics.

The active compounds were extracted from CHEMBL [45] and BindingDB [46] with a previous filtration of the compounds with an experimental IC_50_ or K_d_ under 5500 nm. For each active molecule, 50 decoys were randomly generated using the Database of Useful Decoys: Enhanced (DUD-E) [47]. The test set was composed of a total of 68 active compounds and 3400 decoys. Several metrics were used to evaluate the quality of the VS protocol. Early recognition metrics, such as the Receiver Operating Characteristic curve (ROC), the Area under the Curve (AUC) and the Enrichment Factor (EF), were calculated using Microsoft excel. The Total Gain (TG), Robust Initial Enhancement (RIE), Boltzmann enhanced discrimination of ROC (BEDROC) and Hit Rate were calculated using the online “Screening Explorer” server [48].

### 2.4. Virtual Screening for the Identification of Potential PknB Binders

The optimized VS protocol obtained in the previous step and the SF GOLD/ASP was applied to three large databases of compounds in search of promising molecules to be tested experimentally as possible PknB inhibitors. The virtual databases selected were: the FDA (U.S. Food and Drug Administration)-approved database, a subset of the ZINC database [49], the Mu.Ta.Lig. Virtual Chemotheca [50] and the French National chemical library (Chimiothèque Nationale) [51].

The ZINC database is a free archive of commercially available compounds, provided by the Irwin and Shoichet Laboratories in the Department of Pharmaceutical Chemistry at the University of California (UCSF), that contains over 230 million compounds. For this study, a set of 3206 FDA-approved compounds was tested. Drug repurposing is a powerful tool in finding new uses to already known and well-characterized drugs as antimicrobial agents, whether used alone or in synergy with other antimicrobials [52]. The Mu.Ta.Lig. Virtual Chemotheca was created from COST Action CA15135 and is composed of molecules already synthetized/isolated and tested during the medicinal chemistry research for lead compounds [50]. A total of 64,804 compounds were screened against PknB. The Chimiothèque Nationale offers a diverse collection of more than 70,000 compounds and 15,000 original and little-tested natural extracts. In this study, a total of 61,640 compounds from the Chimiothèque were screened.

The top compounds of each database were evaluated using pharmacokinetics and pharmacodynamics predictors such as SwissADME [53] and pkCSM [54]. In the top results, the toxic and carcinogenic compounds were filtered out. Compounds with a more positive LogP values were also favored due to their higher lipophilicity, and hence, higher probability of crossing the mycobacterial envelope. A selection of molecules of each database was performed and the selected molecules were moved on to the molecular dynamics and free energy calculation stages.

### 2.5. Molecular Dynamics (MD) Simulations

The Molecular Dynamics (MD) simulations performed in this work were conducted using the Amber18 software package [55]. To assign the molecular mechanics parameters, ANTECHAMBER was used, taking into account the general amber force field [56], with RESP charges calculated using HF/6–31G(d) with Gaussian16 [57]. The protein was described using the Amber14sb force field [A]. The protein–ligand complexes were contained in a box of TIP3P water molecules. The edges of the water box were placed at a distance of at least 12 Å from each atom of the complex. Periodic boundary conditions were used on all MD simulations. Long-range electrostatic interactions were calculated using the particle-mesh Ewald summation method. For the electrostatic and Lennard-Jones interactions, a cut-off value of 10.0 Å was used. All bonds involving hydrogen atoms were constrained using the SHAKE algorithm. This allowed the application of a 2-fs time step.

All solvated ligand–PknB complexes went through a phased minimization procedure with four energy-minimization stages to remove bad contacts and optimize the system prior to the MD simulation. In the first stage (2500 steps), only the positions of the water molecules were optimized, with the remaining parts of the system kept constrained to their initial positions through the application of a harmonic potential (50 kcal mol^−1^ Å^−2^). In the second stage (2500 steps), all hydrogen atoms present in the system were optimized. In the third stage (2500 steps), only the positions of the protein backbone atoms were kept constrained, enabling the optimization of the amino-acid side chains. Finally in the fourth stage (10,000 steps), all constrains were removed, enabling a full optimization of the model. After the energy minimization steps, all complexes were heated from 0 to 310.15 K over 50 ps, using a Langevin thermostat at constant volume (NVT ensemble). They were further equilibrated at 310.15 K during 50 ps to stabilize the density. The production phase was run for 100 ns using an NPT ensemble, with a pressure of 1 bar (Berendsen barostat) and a temperature of 310.15 K (Langevin thermostat). The trajectory analysis was performed using the cpptraj tool [58] and VMD [59]. To produce the high-quality protein figures, PyMOL [60] was used.

### 2.6. MM/GBSA Free Energy Calculations

The MM/PBSA.py [61] script from Amber was used to perform the MM/GBSA calculations. The calculations used the last 40 ns of each MD simulation, with an interval of 100 ps between each structure (a total of 800 structures per complex). A salt concentration of 0.100 mol dm^−3^ was used. To gather information about the contribution of each active-site amino acid residue to the overall free energy, the per-residue free energy decomposition option was used.

### 2.7. Data Analysis

All the data from the docking, re-docking, VS studies and MD simulations results were treated using the average and standard deviation tools from Microsoft Excel. To submit the VS results to the online “Screening Explorer” server, a csv file composed of 3 columns with the Identification of the compounds, the score (ranked from the best to the worst), and the numerical indication of Active (1) or Decoy (0) was created for each SF.

## 3. Results and Discussion

Table 2 compares the RMSD (Å) values for the re-docking of all the PknB protein structures studied obtained with all the SFs used: Vina, CHEMPLP, ASP, ChemScore and GoldScore.

Molecular docking studies were performed to evaluate the effect of the metal ions (Mg^2+^ and Mn^2+^) on the re-docking scores. All scores improved in the presence of magnesium ions (data not shown), indicating that they are indeed crucial for the stabilization of the ATP analogs and other binders.

Generally, the SFs that better reproduced the crystallographic pose were Vina, with an average value of 1.09 Å, followed by ASP, with a value of 1.70 Å. On the other hand, all the other GOLD SFs did not show a good performance (CHEMPLP–2.03 Å, ChemScore 3.22 Å and GoldScore-2.15 Å). ChemScore was the SF that had the most difficulty in the re-docking, which can be explained by the fact that bigger, more flexible ligands, such as ATP analogs, can be more challenging to place correctly [43]. Overall, the RMSD values are below 2 Å across most targets, indicating that there is a general good performance with the different docking programs/SFs. According to the cross-docking results (Supplementary Materials—Tables S2–S6) the target 1O6Y and 3F61 were the best in the cross-docking of all the ligands, across most of the SFs tested.

The structures 3ORI and 3ORL also presented very good scores for all the SFs; however, these structures did not perform as effectively in the re-docking stage (with RMSD values of 2.14 Å and 2.47 Å, respectively). Furthermore, the presence of manganese cations and mutations made them less suitable to move on to the VS of large databases of compounds. Based on these results, the choice of the structures to continue the protocol were 1O6Y and 3F61, which had the best scores in 3 out of the 5 different SFs. The GOLD SFs were chosen to progress to the next stage, due to their observed higher computational efficiencies achieved with similar accuracy levels.

Table 3 compares the ability of all tested GOLD SFs (CHEMPLP, ASP, ChemScore and GoldScore) in discriminating between binders and decoys for both studied structures, using several metrics. The enrichment factor is described as the ratio between the percentage of active compounds in the selected subset and the percentage in the entire database [62]. A high EF in the top 1% means that the scoring function can find more true positives early on, and it means that the scoring function is adequate or better for a specific target. However, this metric is highly dependent on the number of actives and is unable to discriminate between SFs that present actives in the top of the list and SFs that present actives just before the threshold [63]. For these reasons, the robust initial enhancement (RIE) was also considered since it does not depend on the number of actives used and is less susceptible to variations when a small number of actives are used. Comparing the structures studied, the higher EF1% was obtained for structure 1O6Y across all the SFs. However, the ASP scoring function stands out with an EF1% of 3.76.

**Figure 3:**
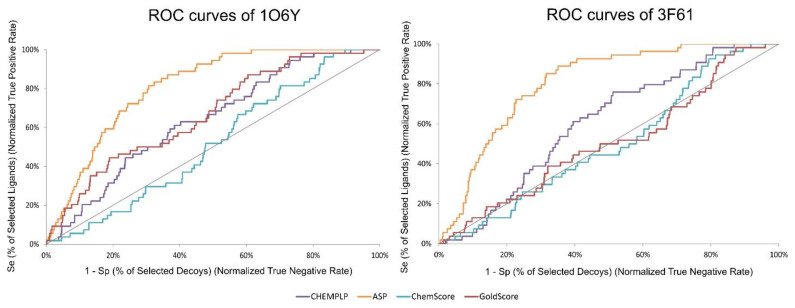
ROC curves obtained for target structures 1O6Y and 3F61 with the different scoring functions used in the active vs. decoys virtual screening evaluation stage.

A ROC curve is obtained by plotting the true positive rate (TPR) against the false positive rate (FPR). In other words, if only true positives are found at the top of the database, it leads to a higher ROC curve and an AUC of 100%. BEDROC is a normalization of the RIE bounded by 0 and 1. TG quantifies the discrimination of actives over decoys attributable to score variations. TG values over 0.25 combined with an AUC over 0.5 indicate a good performance and reproducibility from the VS protocol [48]. In Figure 3, it is possible to visualize the difference in ROC curves between targets and SFs. ASP stands out as the SF that can find more true positives early on (3.76) and as the one that shows a higher AUC (80.9%). ChemScore was the SF that performed the worst in both targets. Since the best performing SF, across all the metrics and for both targets, was ASP, it was this SF that was selected in the virtual screening of large databases of compounds.

**Figure 4:**
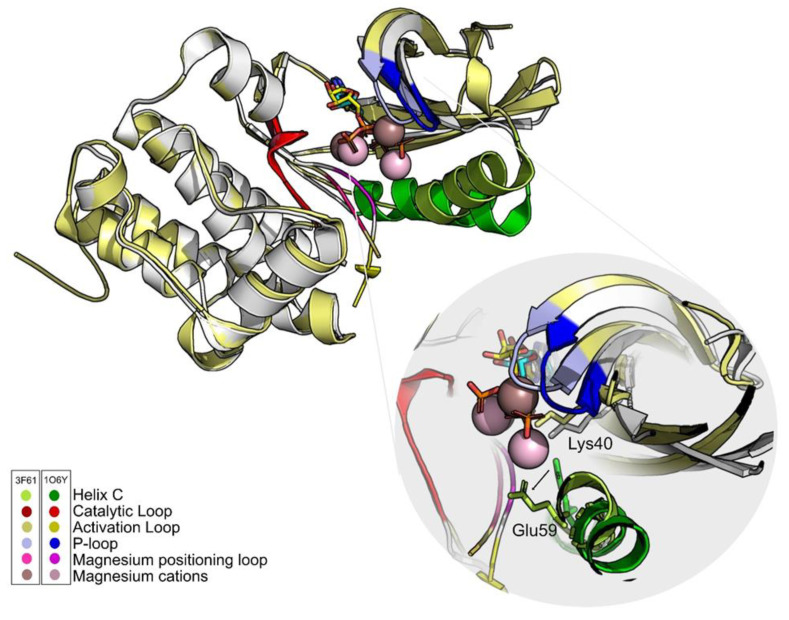
The ATP analogs are shown in cyan (1O6Y) and yellow (3F61) sticks. Differences in the position of the main PknB groups are highlighted. One of the fundamental differences between structures is the position of Glu59. In 3F61, this residue shifts away from the active site and the hydrogen bond with Lys40 does not occur. The only group that maintains its positions in both structures is the catalytic loop (red). Figure was produced using Pymol.

An explanation for the discrepancy of results between the two structures might be the position of the residue Glu59 and the position of the magnesium positioning loop (Figure 4). Some authors defend that the position of this loop is important to bring the helix C to its activated form [64], that is, promoting the contact between Lys40 and Glu59. For this reason, the structure that was chosen to move on to the VS stage was 1O6Y.

From the virtual screening results, a selection of molecules was used to perform MD simulations. These molecules were chosen based on their ranking during the virtual screening procedure and their molecular diversity. For the molecules from the Chemotheca and from Chimiothèque Nationale databases, Lipinski’s rule of five [65,66] was also considered.

**Figure 5:**
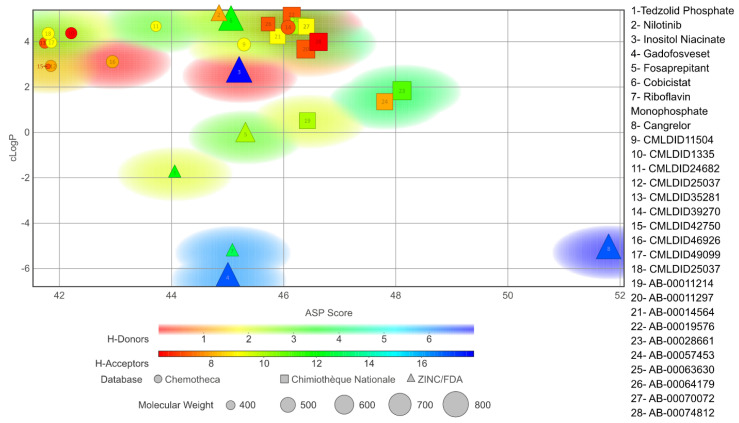
Graph displaying the physico-chemical diversity of the molecules selected for the MD simulations.

The chosen molecules from ZINC/FDA were Inositol Niacinate, Riboflavin Monophosphate, Fosaprepitant, Nilotinib, Gadofosveset, Tedizolid Phosphate, Cobicistat and Cangrelor. From Chemotheca, the chosen molecules were CMLDID11504, CMLDID1335, CMLDID24682, CMLDID25037, CMLDID35281, CMLDID39270, CMLDID42750, CMLDID46926, CMLDID49099 and CMLDID57593. Finally, from Chimiothèque Nationale, the chosen molecules were AB-00011214, AB-00011297, AB-00014565, AB-00019576, AB-00028661, AB-00057453, AB-00063630, AB-00064179, AB-00070072 and AB-00074812. All selected molecules, their scores, and some of their physical–chemical properties can be seen in Figure 5. These properties and figures were obtained using the data visualization program DataWarrior [67]. As a reference molecule, Mitoxantrone was used. Two of the chosen molecules have been reported as active against *M. tuberculosis* or other members of the *Mycobacteriaceae* family. Nilotinib was shown to regulate protective innate immune responses against intracellular *Mycobacterium bovis* and *Mycobacterium avium* [68]. Tedizolid Phosphate was reported to have powerful bactericidal capabilities against *M. avium* [69] and showed promising capabilities against the *Mycobacterium abscessus* complex [70]. Furthermore, Tedizolid Phosphate has also been reported to be active against *M. tuberculosis* [71,72].

To validate the molecular docking results, the structural stability of the protein–ligand complex was evaluated, and MM/GBSA calculations and molecular dynamics simulations were carried out. For each molecule, in complex with 1O6Y, 100 ns of simulation was performed, starting from the posed predicted from docking. To evaluate the structural stability, multiple properties were calculated for the last 40 ns of simulation for each ligand–protein complex, and these are presented in Table 4.

**Figure 6:**
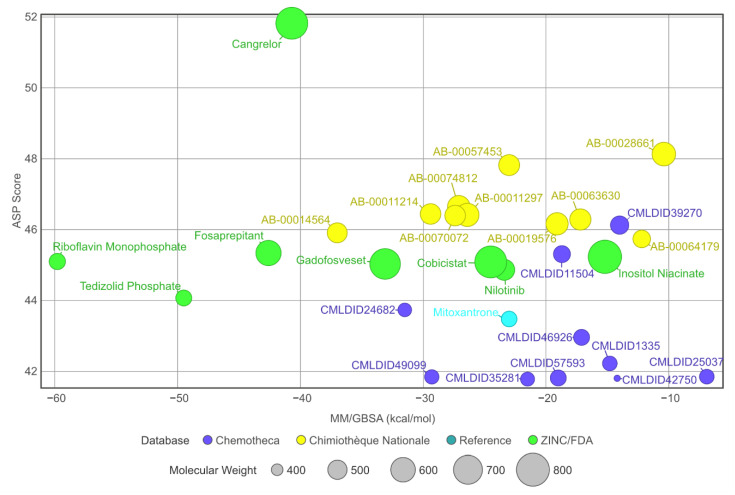
Graph displaying and comparing the results of the free-energy calculation with the results from the virtual screening. The green ligands are the ligands obtained from the ZINC/FDA database, the blue ligands are part of Chemotheca and the yellow ligands are from Chimiothèque Nationale. The reference ligand is in cyan.

When comparing to the initial docking pose, 13 of the 28 ligands had an RMSD value lower than 2 Å (for further information, see Supplementary Materials—Figures S1–S3). Even in the cases where the RMSD value was higher, the standard deviation remained low. This suggests an induced-fit adjustment to the binding pocket of PknB. All molecules remained bound to the protein in the predicted binding location. The stability of the complexes is further supported by the stable SASA values observed during the last 40 ns of simulation. Considering that all protein–ligand complexes were stable, MM/GBSA calculations could be performed. The results are presented in Table 5 and a comparison of the MM/GBSA results with the virtual screening scores is displayed in Figure 6.

A total of 15 of 28 ligands performed equal or better than the reference ligand. From these, two were from Chemotheca, six were from Chimiothèque Nationale and seven were from ZINC/FDA. Between these ligands, these MM/GBSA values varied between −23.0 ± 0.1 and −59.8 ± 0.3 kcal/mol, compared to Mitoxantrone’s −23.0 ± 0.2 kcal/mol. The best ligand from Chemotheca was CMLDID24682, with a MM/GBSA value of −31.5 ± 0.1 kcal/mol. The molecule from Chimiothèque Nationale with the best performance was AB-00014564, with a result of −37.0 ± 0.1 kcal/mol. Lastly, the ligand from ZINC/FDA with the best results was Riboflavin Monophosphate with an MM/GBSA value of −59.8 ± 0.3 kcal/mol.

**Figure 7:**
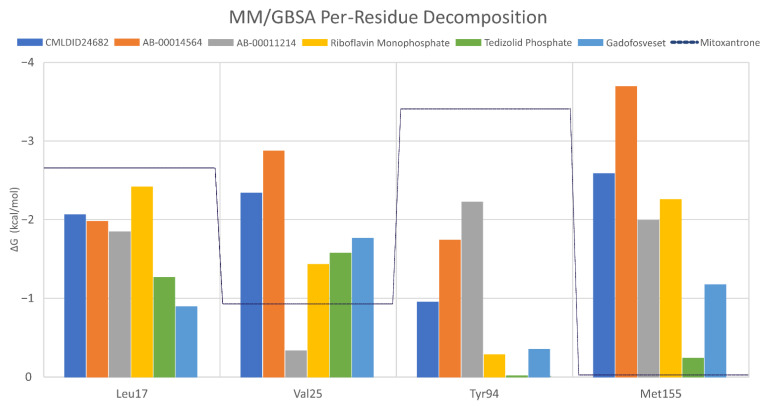
Per-residue decomposition of the free energy calculations using MM/GBSA for the best performing ligands.

To further analyze the affinity between the best performing ligands and the receptor, the overall binding free energy was decomposed into the contribution of each residue. The residues that had, in general, a bigger contribution are represented in Figure 7. The six top performing ligands were chosen for this analysis, one from Chemotheca (CMLDID24682), two from Chimiothèque Nationale (AB-00014564 and AB-00011214) and three from ZINC/FDA (Table 6). From ZINC/FDA, the molecules chosen were Riboflavin Monophosphate, Tedizolid phosphate and Gadofosveset trisodium.

Overall, the PknB residues that had higher contributions for binding free energy were Leu17, Val26, Tyr94 and Met155. For CMLDID24682, Val 25 and Met155 had the greater contributions, with ∆G values of −2.3 ± 0.3 and −2.6 ± 0.4 kcal/mol, respectively. The residues with the higher contributions were maintained for AB-00014564, with a ∆G value of −2.9 ± 0.4 kcal/mol for Val 25 and of −3.7 ± 0.8 kcal/mol for Met155. In the case of AB-00011214, Met155 remained one of the more important residues, having a ∆G value of −2.0 ± 0.7 kcal/mol, and was joined by Tyr94, which had a ∆G value of −2.2 ± 0.5 kcal/mol. For the binding of the first of the FDA molecules, Riboflavin Monophosphate, the greater contribution came from Leu17 and Met155, presenting ∆G values of −2.4 ± 0.5 and −2.3 ± 0.4 kcal/mol, respectively. As for the binding of Tedizolid Phosphate, the greater contribution came from Val25, with a ∆G value of −1.6 ± 0.5 kcal/mol. The same was observed for the final ligand, Gadofosveset, in which Val25 presented the higher contribution to the binding free energy with a decomposed ∆G value of 1.8 ± 0.3 kcal/mol.

**Figure 8:**
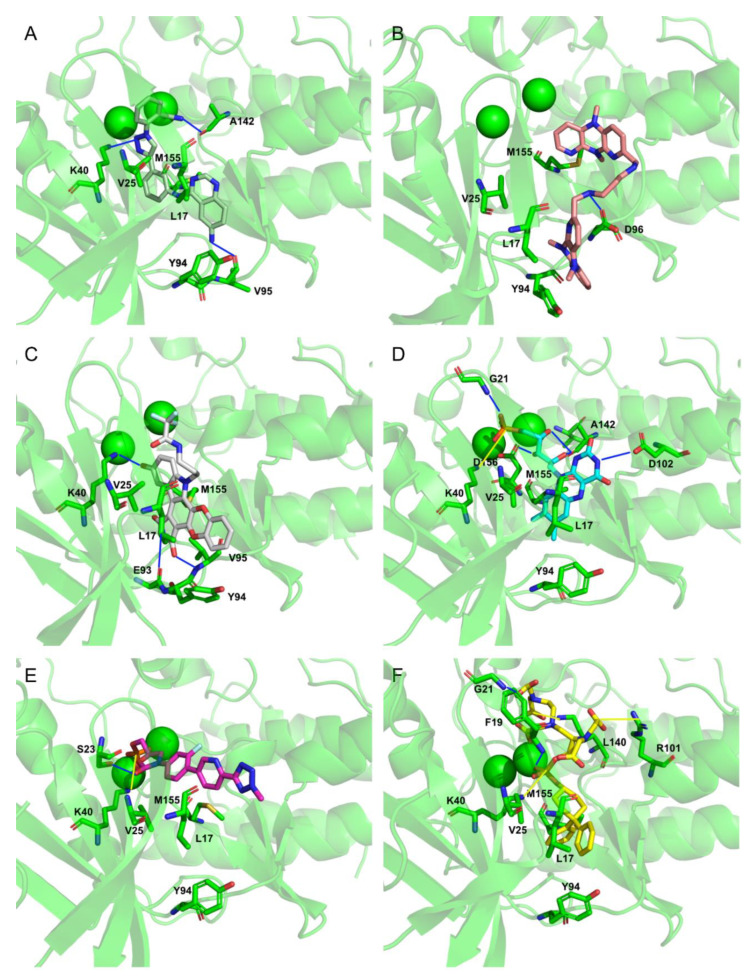
Representation of the Interactions of CMLDID2468 (**A**), AB-00011214 (**B**), AB-00014564 (**C**), Riboflavin Monophosphate (**D**), Tedizolid Phosphate (**E**) and Gadofosvese (**F**) bound to PknB. Figures were produced using Pymol.

**Figure 9:**
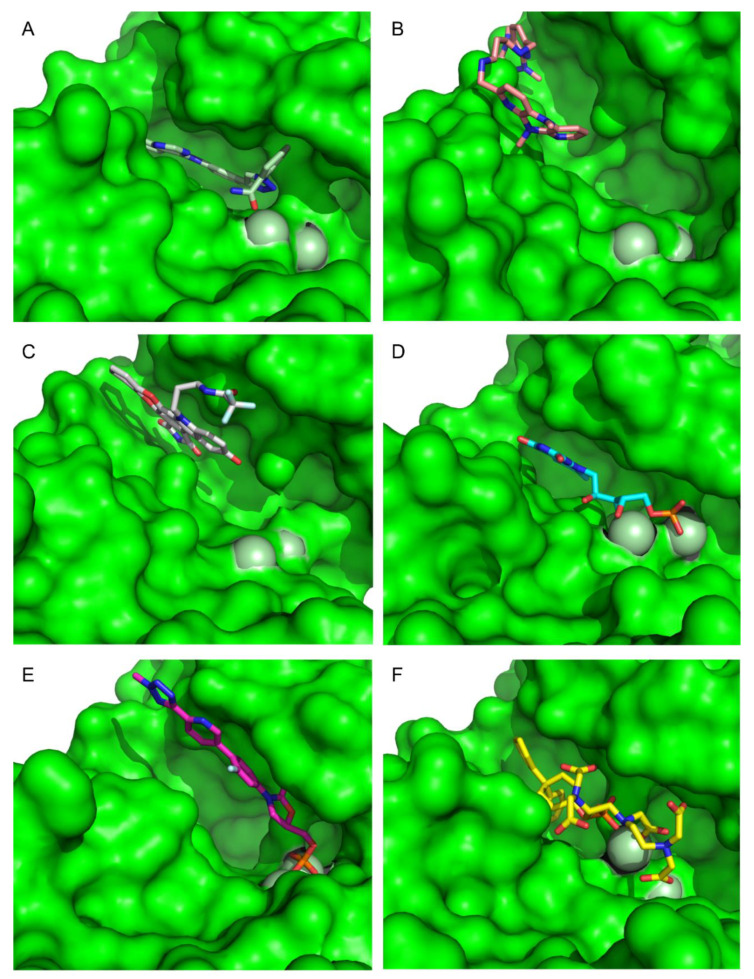
Surface representation of CMLDID2468 (**A**), AB-00011214 (**B**), AB-00014564 (**C**), Riboflavin Monophosphate (**D**), Tedizolid Phosphate (**E**) and Gadofosvese (**F**) bound to PknB. Figures were produced using Pymol.

To extract the most representative structure from the MD simulations, the cluster command from cpptraj was used [59]. The six best performing molecules and their interactions are represented in Figure 8 and Figure 9. The represented interactions were obtained by using the Protein–Ligand Interaction Profiler [76].

During the interaction of CMLDID2468 with PknB, multiple hydrogen bonds were established. Lys40 interacted with the Triazine group, and Val95 interacted with the nitrogen from the terminal aniline group. Lastly, Ala144 interacted with the nitrogen in the other terminal group. Throughout the simulation, there was a shift in the position of the magnesium ions and the carboxamide region of the molecule was stabilized by one of them. The P-loop accompanied the shift of the cations and moved toward the front, and Glu59 was oriented toward the second Mg^2+^.

AB-00011214 only formed one hydrogen bond with PknB. Asp96 interacted with one of the secondary amine groups. Throughout the simulation, there was a shift in the position of the P-loop of PknB opening the catalytic site, increasing the distance between Lys40 and Glu59. This occurred due to the structure of the ligand. It was a large and flexible molecule and as it unfolded in the catalytic site, it triggered a shift in the position of the residues of the nucleotide-binding area as well as the Mg cations.

As for AB-00014564, this ligand interacted with PknB with three hydrogen bonds. Lys40 interacted with the phenol group. Glu93 and Val95 interacted with the Pyrrolidine-2,5-diol group, with the nitrogen and one of the oxygens, respectively. The molecule occupied a higher position in nucleotide-binding area of the PknB binding pocket, causing a shift in the P-loop region, the Mg2^+^ ions and magnesium position loop, toward the C-lobe. The pocket, however, maintained its closed configuration.

Riboflavin Monophosphate established multiple interactions. Gly21 formed a hydrogen bond with the phosphate group. Lys40 formed a salt bridge with the same phosphate group. Asp102 established a hydrogen bond with one of the nitrogens from the Imidazolidine-2,4-diol group. Finally, Ala142 and Asp156 formed hydrogen bonds with the three oxygens from the main chain. The phosphate groups of this compound interacted with the magnesium cations in the same way as the ATP analogs, and hence, there was no significative difference in the positions of the P-loop and magnesium positioning loop.

The Tedizolid Phosphate bond to PknB formed a hydrogen bond between Ser23 and the oxygen from the Hydroxyoxazolidin group. Lys40 established a salt bridge with the phosphate group. The phosphate group of Tedizolid Phosphate also interacted with the Mg^2+^ in the same way as the ATP analogs; however, the ring portion of compound was not in the same place as the adenine moiety of ATP would be, leading to a shift of the activation and catalytic loops.

Finally, Gadofosveset established two hydrogen bonds with PknB. Phe19 and Gly21 interacted with two of the ligand’s carboxylic acids. In addition, Arg101 and Lys140 formed salt bridges with two other carboxylic acids. Finally, Lys40 formed a salt bridge with the phosphate group. Throughout the simulation, there were no significant differences in the position of the P-loop. However, the phosphate group, which was present in the middle of the ligand, was stabilized by the Mg cations, so it was oriented in a way that was closer to the cations than to where the adenine moiety of ATP would have been. This also led to a shift in the position of the activation and catalytic loops.

## 4. Conclusions

In this article, an in silico methodology is described for the discovery of new mycobacterial serine/threonine-protein kinase, PknB, inhibitors. From the six compounds identified as potential inhibitors, only one (AB-00011214) was able to turn the active site from a closed to an open conformation, with a shift in the position of the residue Glu59. In the simulations of the other five compounds, there was a slight shift of the Glu59, but only toward one of the Mg cations; this was not enough to lead to an open conformation. Two of the FDA compounds (Tedizolid Phosphate and Gadofosveset) caused significative changes in the position of the activation and catalytic loops, with Tedizolid showing intracellular antimicrobial activity against Mtb. Of course, the precise kinetic effect of these structural modifications is still not clear; however, these results can provide clues toward a better understanding of the inhibitory mechanism. Further in vitro and in vivo experimental studies on the drugs described should be considered to evaluate their ability to cross the bacterial envelope and confirm their actual potential as anti-tuberculosis agents, not only against Mtb but also toward resistant strains.

In conclusion, the data presented here demonstrate that this multi-level computational approach accurately predicted the binding position of several crystallographic compounds and identified six potential strong candidates for PknB inhibition. It also provided solid structural information on the protein–ligand interactions that can be used to design more specific and effective derivatives.

## Figures and Tables

**Table 1:** Available structures of *M. tuberculosis* PknB on PDB.

Code	Ligand	Description	Resolution (Å)	Metal ion	Mutation	Ref.
1MRU	ATP analog	Chains A, B	3.00	Mg^2+^	0	[17]
1O6Y	ATP analog	Catalytic Domain	2.20	Mg^2+^	0	[16]
2FUM	Antagonist	Chains A, B, C, D	2.89	-	0	[18]
3F61	ATP analog	Catalytic Domain	1.80	Mg^2+^	2 (L33D/V222D)	[31]
3F69	Antagonist	Chains A, B	2.80	-	3 (L36D/M148L/M158V)	[31]
3ORI	ATP analog	Chains A, B, C, D	2.00	Mn^2+^	1 (L33D)	[32]
3ORK	ATP analog	Catalytic Domain	1.60	Mn^2+^	1 (L33D)	[32]
3ORL	ATP analog	Catalytic Domain	2.90	Mn^2+^	1 (L33D)	[32]
3ORM	ATP analog	Catalytic Domain	2.50	Mn^2+^	1 (D76A)	[32]
3ORO	ATP analog	Catalytic Domain	1.90	-	1 (L33D)	[32]
3ORP	ATP analog	Catalytic Domain	2.10	-	1 (L33D)	[32]
3ORT	ATP analog	Catalytic Domain	1.90	-	1 (L33D)	[32]
5U94	Antagonist	Catalytic Domain	2.20	Mg^2+^	0	[33]
6B2P	Antagonist	Catalytic Domain	3.01	-	0	[34]
6I2P	ATP analog	Bound to GarA ^1^	2.37	Mg^2+^	1 (L33E)	[35]

^1^ Glycogen accumulation regulator (GarA).

**Table 2:** Re-docking RMSD (Å) values for the all the PknB structures for all the docking programs used.

PDB Code	Ligand	Vina	CHEMPLP	ASP	ChemScore	GoldScore	Average per Target ^1^
1MRU	ATP analog	0.57	4.98	2.42	6.77	2.35	3.42 ± 2.45
1O6Y	ATP analog	0.82	1.38	1.43	2.43	1.13	1.44 ± 0.61
2FUM	Antagonist	0.84	2.01	1.94	2.40	2.37	1.91 ± 0.63
3F61	ATP analog	0.18	1.53	1.72	5.14	0.63	1.84 ± 1.95
3F69	Antagonist	0.50	0.31	0.56	0.81	0.81	0.60 ± 0.21
3ORI	ATP analog	0.51	3.33	0.93	2.77	3.33	2.17 ± 1.36
3ORK	ATP analog	3.25	1.61	1.46	3.63	2.11	2.41 ± 0.97
3ORL	ATP analog	1.17	2.79	2.31	3.00	3.07	2.47 ± 0.78
3ORM	ATP analog	1.68	3.5	1.49	3.06	3.9	2.73 ± 1.08
3ORO	ATP analog	0.74	1.37	2.8	2.85	0.85	1.72 ± 1.03
3ORP	ATP analog	1.16	2.58	2.37	2.33	0.5	1.79 ± 0.91
3ORT	ATP analog	0.22	2.55	1.79	2.58	2.33	1.89 ± 0.99
5U94	Antagonist	0.25	0.04	1.14	5.97	0.60	1.60 ± 2.47
6B2P	Antagonist	2.81	1.40	1.88	3.46	6.00	3.11 ± 1.80
6I2P	ATP analog	1.68	1.10	1.35	1.13	2.23	1.50 ± 0.47
Average per SF ^2^		1.09 ± 0.92	2.03 ± 1.28	1.70 ± 0.61	3.22 ± 1.63	2.15 ± 1.52	

^1^ *n* = 5; ^2^ *n* = 15.

**Table 3:** Evaluation metrics for Active vs. Decoys virtual screening results for the selected targets.

	1O6Y					3F61				
	EF 1%	AUC%	TG	RIE	BEDROC	EF 1%	AUC	TG	RIE	BEDROC
CHEMPLP	1.88	63.7	0.18	1.44	0.08	0.00	60.6	0.12	0.65	0.04
ASP	3.76	80.9	0.40	3.19	0.19	1.88	80.6	0.40	2.87	0.17
ChemScore	1.88	51.4	0.02	0.73	0.04	0.00	50.1	0.01	0.65	0.04
GoldScore	1.88	66.7	0.24	2.58	0.15	0.00	50.6	0.02	1.00	0.06

**Table 4:** Average RMSD values (Å), RMSF (Å), average SASA (Å^2^), percentage of potential ligand SASA buried and an average number of hydrogen bonds for the ligands for the last 40 ns of the simulation of the PknB–ligand complexes.

Database	Molecules	Average RMSD (Å) ^1^	RMSF (Å)	Average SASA (Å^2^) ^1^	Percentage of Potential Ligand SASA Buried (%)	Average # H-Bonds ^1^
Mu.Ta.Lig Chemotheca	CMLDID11504	3.56 ± 0.67	1.4	199 ± 65	53 ± 3	0.3
	CMLDID1335	1.74 ± 0.41	1.2	395 ± 43	51 ± 5	0.4
	CMLDID24682	0.69 ± 0.12	0.3	194 ± 172	57 ± 3	1.5
	CMLDID35281	2.26 ± 0.73	1.4	374 ± 119	50 ± 4	0.3
	CMLDID39270	2.44 ± 0.60	1.8	230 ± 48	52 ± 3	0.3
	CMLDID42750	1.58 ± 0.44	1.4	182 ± 48	58 ± 3	0.3
	CMLDID46926	1.88 ± 0.54	1.4	235 ± 49	55 ± 3	0.4
	CMLDID49099	1.29 ± 0.32	0.9	265 ± 30	56 ± 6	1
	CMLDID57593	1.93 ± 0.40	1.1	303 ± 27	45 ± 3	0.4
	CMLDID25037	2.17 ± 0.30	1.2	284 ± 39	42 ± 4	0.2
Chimiothèque Nationale	AB-00011214	2.92 ± 0.26	1.3	394 ± 116	61 ± 3	0.9
	AB-00011297	1.48 ± 0.36	1.1	570 ± 143	51 ± 4	0.5
	AB-00014564	2.47 ± 0.57	1.1	187 ± 47	48 ± 5	2
	AB-00019576	2.39 ± 0.47	1.6	413 ± 165	45 ± 6	0.6
	AB-00028661	3.75 ± 0.47	1.7	448 ± 47	53 ± 4	1.4
	AB-00057453	2.50 ± 0.80	1.8	338 ± 68	48 ± 6	0.6
	AB-00063630	2.03 ± 0.34	1.1	239 ± 38	59 ± 4	0.6
	AB-00064179	1.87 ± 0.49	1	317 ± 33	49 ± 4	0.7
	AB-00070072	1.15 ± 0.37	0.8	328 ± 26	50 ± 3	0.1
	AB-00074812	3.91 ± 1.18	2.9	542 ± 101	63 ± 3	1.3
ZINC/ FDA	Inositol Niacinate	1.77 ± 0.19	1	624 ± 135	50 ± 5	0.2
	Riboflavin Monophosphate	0.70 ± 0.17	0.3	188 ± 31	49 ± 4	0.9
	Fosaprepitant	1.99 ± 0.36	0.8	408 ± 67	57 ± 2	0.3
	Nilotinib	2.51 ± 1.34	2.2	325 ± 69	50 ± 3	0.3
	Gadofosveset	3.96 ± 0.40	2	478 ± 51	53 ± 2	3.6
	Tedizolid Phosphate	1.33 ± 0.34	0.9	266 ± 67	51 ± 4	0.6
	Cobicistat	3.89 ± 1.29	2.9	607 ± 82	53 ± 5	0.7
	Cangrelor	2.27 ± 0.27	1.2	388 ± 216	57 ± 5	0.2
Reference	Mitoxantrone	1.94 ± 0.30	1.3	401 ± 116	62 ± 3	1.3

^1^ n = 2000 frames.

**Table 5:** Results for the MM/GBSA calculations on the selected molecules and all their components.

		MM		GBSA		∆G_binding_
Database	Ligand	E_vdw_ (kcal/mol)	E_el_ (kcal/mol)	E_SURF_ (kcal/mol)	E_GB_ (kcal/mol)	MM/GBSA (kcal/mol)
Mu.Ta.Lig Chemotheca	CMLDID11504	−38.8 ± 0.2	−77.6 ± 0.3	−5.8 ± 0.0	103.5 ± 0.3	−18.7 ± 0.2
	CMLDID1335	−19.3 ± 0.2	−265.0 ± 2.0	−2.5 ± 0.0	272.1 ± 1.9	−14.8 ± 0.2
	CMLDID24682	−46.1 ± 0.1	−111.4 ± 0.2	−6.8 ± 0.0	132.8 ± 0.2	−31.5 ± 0.1
	CMLDID35281	−29.6 ± 0.1	−115.6 ± 1.0	−3.2 ± 0.1	126.9 ± 0.9	−21.5 ± 0.1
	CMLDID39270	−37.4 ± 0.2	−79.7 ± 0.4	−5.4 ± 0.0	108.5 ± 0.3	−14.0 ± 0.2
	CMLDID42750	−37.4 ± 0.2	−58.7 ± 0.2	−5.4 ± 0.0	87.3 ± 0.2	−14.2 ± 0.1
	CMLDID46926	−39.2 ± 0.2	−15.9 ± 0.3	−4.8 ± 0.0	42.8 ± 0.4	−17.1 ± 0.2
	CMLDID49099	−39.2 ± 0.1	−160.8 ± 0.5	−5.3 ± 0.0	176.0 ± 0.5	−29.3 ± 0.1
	CMLDID57593	−29.6 ± 0.1	−2.6 ± 0.1	−3.1 ± 0.0	16.3 ± 0.1	−19.0 ± 0.1
	CMLDID25037	−31.7 ± 0.2	−81.8 ± 0.4	−5.1 ± 0.0	111.8 ± 0.3	−6.9 ± 0.2
Chimiothèque Nationale	AB-00011214	−43.4 ± 0.1	−244.6 ± 0.8	−5.2 ± 0.0	263.7 ± 0.7	−29.4 ± 0.2
	AB-00011297	−40.1 ± 0.2	−114.1 ± 0.4	−4.1 ± 0.0	131.9 ± 0.4	−26.4 ± 0.1
	AB-00014564	−49.2 ± 0.2	−41.5 ± 0.3	−6.1 ± 0.0	59.9 ± 0.2	−37.0 ± 0.1
	AB-00019576	−45.8 ± 0.1	−73.7 ± 0.3	−6.8 ± 0.0	107.1 ± 0,2	−19.1 ± 0.2
	AB-00028661	−31.4 ± 0.2	−197.8 ± 0.7	−5.5 ± 0.0	224.3 ± 0.7	−10.4 ± 0.2
	AB-00057453	−37.8 ± 0.2	−219.4 ± 0.8	−4.3 ± 0.0	238.5 ± 0.7	−23.0 ± 0.1
	AB-00063630	−42.3 ± 0.1	−70.2 ± 0.3	−6.6 ± 0.0	101.9 ± 0.2	−17.2 ± 0.1
	AB-00064179	−33.2 ± 0.1	−55.0 ± 0.3	−4.8 ± 0.0	80.8 ± 0.2	−12.2 ± 0.1
	AB-00070072	−42.7 ± 0.1	7.6 ± 0.1	−4.8 ± 0.0	12.5 ± 0.1	−27.4 ± 0.1
	AB-00074812	−30.1 ± 0.2	−340.0 ± 1.3	−4.6 ± 0.0	347.6 ± 1.2	−27.1 ± 0.2
ZINC/ FDA	Inositol Niacinate	−35.7 ± 0.2	−5.7 ± 0.3	−4.6 ± 0.0	30.8 ± 0.3	−15.2 ± 0.1
	Riboflavin Monophosphate	−16.1 ± 0.2	−584.8 ± 0.9	−6.5 ± 0.0	547.7 ± 0.8	−59.8 ± 0.3
	Fosaprepitant	2.4 ± 0.2	−486.5 ± 0.7	−3.6 ± 0.0	445.1 ± 0.6	−42.6 ± 0.3
	Nilotinib	−38.4 ± 0.3	−17.9 ± 0.5	−5.1 ± 0.0	38.0 ± 0.6	−23.4 ± 0.2
	Gadofosveset	−25.0 ± 0.3	−518.5 ± 1.7	−7.1 ± 0.0	517.4 ± 1.6	−33.1 ± 0.3
	Tedizolid Phosphate	−6.6 ± 0.2	−565.6 ± 0.8	−4.1 ± 0.0	526 ± 0.7	−49.5 ± 0.3
	Cobicistat	−39.6 ± 0.2	−115.2 ± 0.6	−5.2 ± 0.0	135.5 ± 0.6	−24.5 ± 0.2
	Cangrelor	−58.5 ± 0.1	−86.7 ± 0.3	−8.2 ± 0.0	112.8 ± 0.3	−40.7 ± 0.2
Reference	Mitoxantrone	−31.9 ± 0.2	−295.3 ± 1.1	−4.2 ± 0.0	308.4 ± 1.0	−23.0 ± 0.2

**Table 6:** Best performing molecules from the ZINC/FDA database.

Drug Name	Description	Structure
Riboflavin Monophosphate	Riboflavin Monophosphate is a form of vitamin B2 used to restore riboflavin in anaemia, migraine, alcoholism, and hyperhomocysteinemia [73].	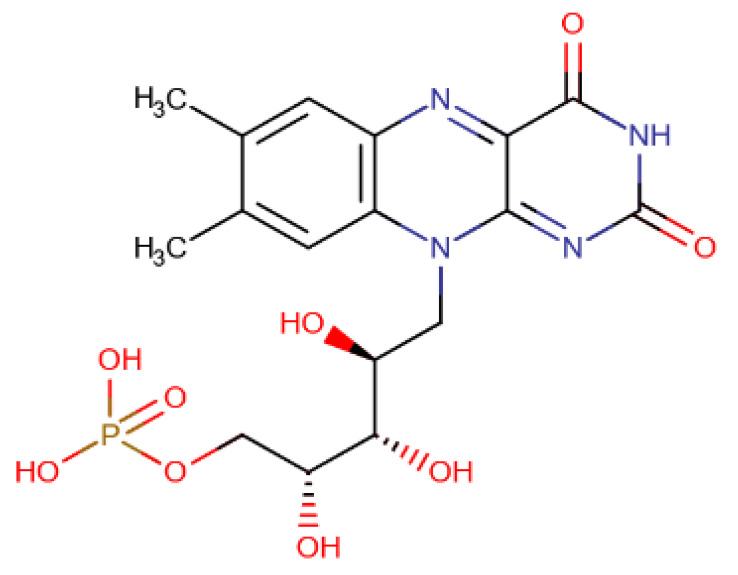
Tedizolid phosphate	Tedizolid phosphate is a member of the oxazolidinone class of antibiotics. It is effective in the treatment of certain Gram-positive bacterial infections. It works by inhibiting protein synthesis by bacterial ribosomes [74].	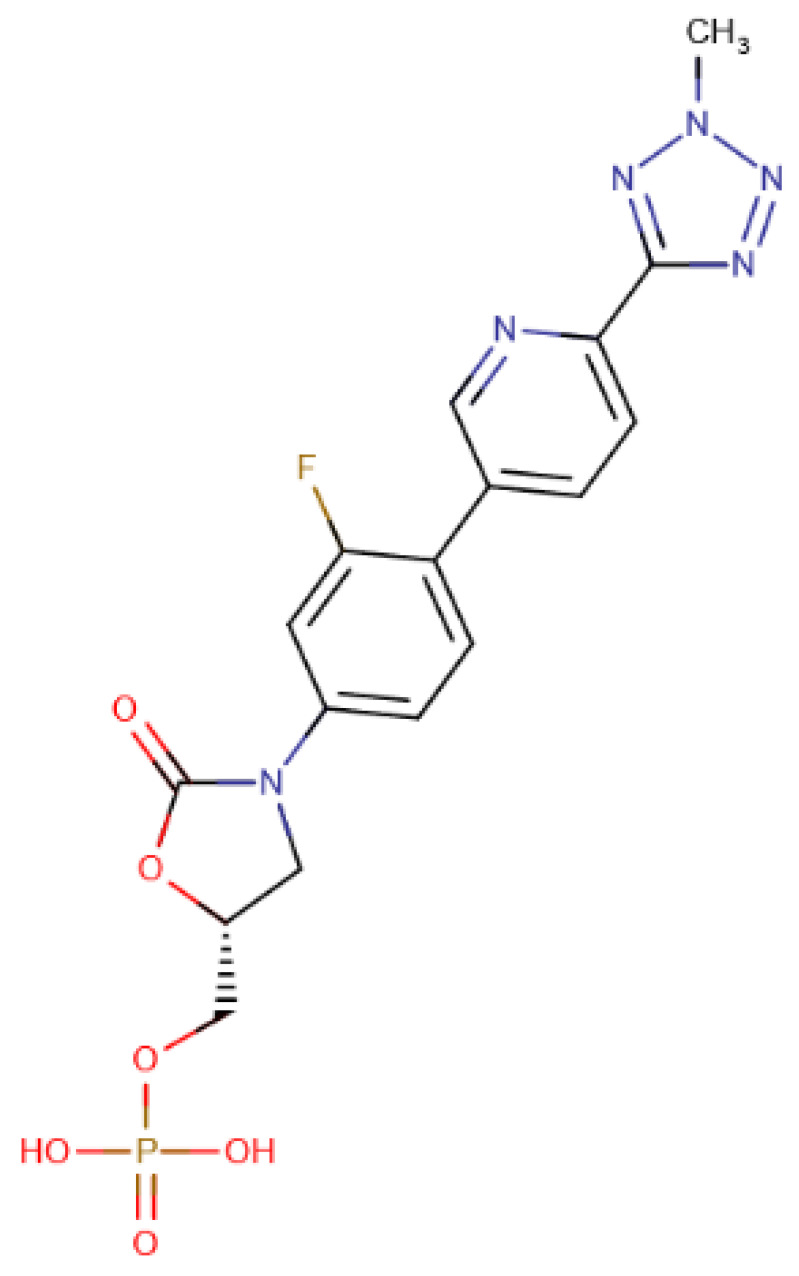
Gadofosveset	Gadofosveset trisodium is an intravenous contrast agent used with magnetic resonance imaging [75].	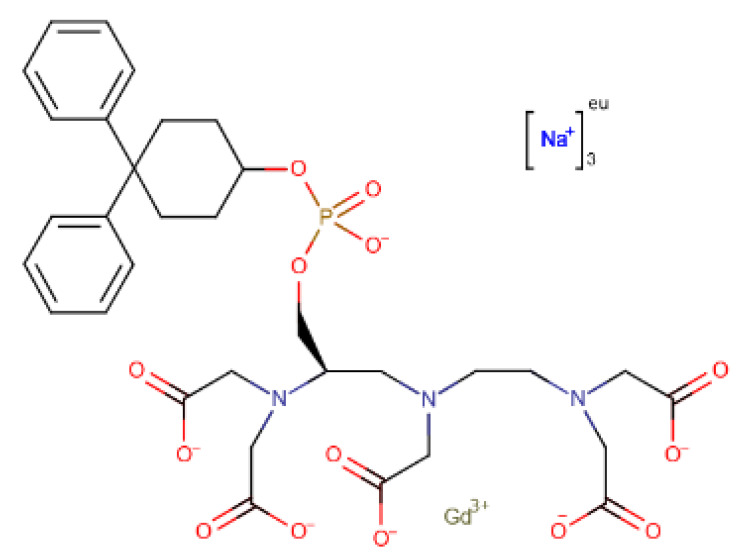

## Supplementary Materials

The following are available online, Figure S1: Root mean square deviation plots for the selected ligands from the ZINC/FDA database, Figure S2: Root mean square deviation plots for the selected ligands from the Mu.Ta.Lig. Virtual Chemotheca, Figure S3: Root mean square deviation plots for the selected ligands from the Chimiothèque Nationale, Table S1: GOLD Re-docking results showing the Impact of the presence of Mg2+ and Mn2+ on the score, Table S2: CHEMPLP Cross-docking score results for all the molecular targets studied. A higher score corresponds to a better affinity, Table S3: ASP score results for all the molecular targets studied. A higher score corresponds to a better affinity, Table S4: ChemScore score results for all the molecular targets studied. A higher score corresponds to a better affinity, Table S5: GoldScore score results for all the molecular targets studied. A higher score corresponds to a better affinity, Table S6: Vina score results for all the molecular targets studied. A more negative score corresponds to a better affinity.

## Sample Availability

Samples of the compounds are available from the databases cited in this work.
