# Supplementary material for: In Silico Identification of Possible Inhibitors for Protein Kinase B (PknB) of Mycobacterium tuberculosis
Source: Molecules. 2021 Oct 12;26(20):6162. doi: 10.3390/molecules26206162 (PMC8541300; doi:10.3390/molecules26206162)
Supplement: Supplementary file 1 [file molecules-26-06162-s001.zip › molecules-1400739-supplementary.pdf]

Supplementary Materials

In silico identification of possible inhibitors for Protein Kinase B (PknB) of *Mycobacterium tuberculosis*

Tatiana F. Vieira[1,2], Fábio G. Martins[1,2], Joel P. Moreira[1,2], Tiago Barbosa[1,2], Sérgio F. Sousa[1,2]

[1] Associate Laboratory i4HB - Institute for Health and Bioeconomy, Faculdade de Medicina, Universidade do Porto, 4200-319 Porto, Portugal
[2] UCIBIO – Applied Molecular Biosciences Unit, BioSIM - Departamento de Biomedicina, Faculdade de Medicina, Universidade do Porto, 4200-319 Porto, Portugal
* Correspondence: sergiofsousa@med.up.pt

Table S1. GOLD Re-docking results showing the Impact of the presence of Mg^{2+} and Mn^{2+} on the score.

**Protein**	**Ligand**	**CHEMPLP**		**GoldScore**		**ChemScore**		**ASP**	
		**w/ cations**	**w/o cations**	**w/ cations**	**w/o cations**	**w/ cations**	**w/o cations**	**w/ cations**	**w/o cations**
1MRU	ATP analog	102.02	70.91	92.11	69.20	33.42	6.92	48.65	44.82
1O6Y	ATP analog	134.40	74.35	105.35	90.90	33.36	5.65	54.16	44.74
3F61	ATP analog	115.63	72.08	100.44	80.97	36.07	13.86	49.52	41.72
3ORI	ATP analog	139.92	73.17	139.56	72.30	36.06	-2.27	62.47	34.46
3ORK	ATP analog	119.28	75.46	131.61	98.06	24.97	13.56	48.17	43.74
3ORL	ATP analog	135.47	84.17	130.32	113.70	31.02	13.94	50.12	46.59
3ORM	ATP analog	104.44	75.35	105.90	95.74	16.76	4.36	52.64	40.45
5U94	Antagonist	101.15	94.94	90.01	90.91	39.67	38.11	37.59	38.17
6I2P	ATP analog	100.40	70.95	76.97	79.60	19.66	4.30	42.42	37.83

Table S2. CHEMPLP Cross-docking score results for all the molecular targets studied. A higher score corresponds to a better affinity.

**CHEMPLP**	**1MRU**	**1O6Y**	**2FUM**	**3F61**	**3F69**	**3ORI**	**3ORK**	**3ORL**	**3ORM**	**3ORO**	**3ORP**	**3ORT**	**5U94**	**6B2P**	**6I2P**
**ATP analog**	111.82	129.30	70.90	128.19	65.63	121.63	114.82	133.42	106.40	84.22	79.81	82.10	108.03	72.76	93.38
**ATP analog**	117.89	139.34	68.44	139.12	126.35	122.27	124.66	133.72	107.99	94.13	98.43	84.04	102.68	75.80	102.33
**Antagonist**	85.40	96.17	91.89	91.65	92.78	85.28	85.78	89.42	94.27	86.27	82.56	90.27	100.31	83.81	87.99
**ATP analog**	99.84	120.88	64.96	113.05	62.79	104.43	92.96	118.50	98.40	95.71	93.94	85.48	105.29	63.33	86.53
**Antagonist**	84.52	87.25	69.96	78.96	94.60	75.86	74.11	74.03	79.28	75.62	65.80	72.10	81.97	70.91	68.35
**Antagonist**	67.54	88.27	67.11	75.63	72.01	80.85	73.10	68.54	85.09	82.31	72.24	60.10	101.34	72.27	73.72
**Antagonist**	64.79	69.13	63.70	72.44	72.17	71.21	73.10	72.00	65.58	62.02	61.81	62.47	69.03	62.33	61.81
**Average**	90.26	104.33	71.00	99.86	83.76	94.50	91.22	98.52	91.00	82.90	79.23	76.65	95.52	71.60	82.02

Table S3. ASP score results for all the molecular targets studied. A higher score corresponds to a better affinity.

ASP	1MRU	1O6Y	2FUM	3F61	3F69	3ORI	3ORK	3ORL	3ORM	3ORO	3ORP	3ORT	5U94	6B2P	6I2P
**ATP analog**	49.27	52.85	31.17	54.81	34.92	54.03	50.85	47.50	51.42	44.25	44.36	45.61	47.06	32.50	43.64
**ATP analog**	50.34	54.17	32.49	57.13	40.20	55.41	51.24	47.47	51.23	48.27	46.01	46.25	49.95	33.47	40.76
**Antagonist**	44.72	43.33	43.31	43.58	41.93	42.89	41.39	41.34	42.64	42.18	42.74	38.60	45.15	40.99	39.15
**ATP analog**	45.08	48.94	30.52	49.11	36.49	49.71	45.63	46.18	47.88	40.82	39.85	38.15	42.68	41.00	40.15
**Antagonist**	40.27	41.00	32.62	30.71	41.99	35.63	30.60	32.49	37.43	35.90	34.65	28.87	37.63	34.93	34.91
**Antagonist**	29.08	37.17	29.74	30.21	30.30	32.42	28.41	28.62	32.01	28.89	29.40	26.33	39.42	31.05	25.98
**Antagonist**	33.22	32.96	33.00	31.61	33.69	26.95	29.83	35.05	32.43	32.21	30.47	30.96	35.62	32.21	29.59
**Average**	41.71	44.35	33.26	42.45	37.07	42.43	39.71	39.81	42.15	38.93	38.21	36.40	42.50	35.16	36.31

Table S4. ChemScore score results for all the molecular targets studied. A higher score corresponds to a better affinity.

ChemScore	1MRU	1O6Y	2FUM	3F61	3F69	3ORI	3ORK	3ORL	3ORM	3ORO	3ORP	3ORT	5U94	6B2P	6I2P
**ATP analog**	27.72	37.99	6.61	34.38	2.60	16.18	25.91	30.26	14.14	9.13	10.85	6.45	19.67	4.04	20.17
**ATP analog**	26.82	33.42	31.84	35.45	2.67	19.97	27.32	29.71	14.84	13.96	11.06	8.49	13.79	5.00	20.41
**Antagonist**	42.99	33.78	31.86	37.34	30.17	32.20	34.25	29.23	32.57	30.40	29.36	23.36	33.95	27.33	30.27
**ATP analog**	30.38	38.56	8.89	34.36	7.41	24.87	29.31	34.67	22.51	18.93	15.02	12.46	20.64	8.95	20.53
**Antagonist**	44.22	42.62	31.22	36.71	38.36	37.00	35.74	30.20	33.39	34.51	28.87	26.23	39.22	31.03	30.84
**Antagonist**	40.91	39.48	31.00	32.76	29.58	35.32	29.60	34.08	30.13	30.18	33.47	30.31	40.61	39.21	30.52
**Antagonist**	39.64	39.78	24.79	38.25	26.51	38.88	40.46	43.51	35.37	30.65	28.67	26.45	38.81	23.44	37.03
**Average**	36.10	37.95	23.74	35.61	19.61	29.20	31.80	33.09	26.14	23.97	22.47	19.11	29.53	19.86	27.11

Table S5. GoldScore score results for all the molecular targets studied. A higher score corresponds to a better affinity.

GoldScore	1MRU	1O6Y	2FUM	3F61	3F69	3ORI	3ORK	3ORL	3ORM	3ORO	3ORP	3ORT	5U94	6B2P	6I2P
**ATP analog**	95.45	106.61	84.58	120.61	78.90	132.64	134.75	132.17	124.27	110.18	112.25	101.62	97.66	98.16	78.17
**ATP analog**	108.12	115.14	108.74	119.12	104.28	141.47	130.46	129.33	113.22	108.12	113.53	95.72	102.71	99.09	80.14
**Antagonist**	71.68	92.77	90.21	75.49	80.70	94.32	76.58	86.46	90.05	84.12	77.92	79.67	85.77	81.54	75.26
**ATP analog**	79.75	95.88	76.84	92.89	65.77	117.31	117.30	122.50	98.89	95.61	101.96	92.20	84.60	73.69	69.75
**Antagonist**	74.56	78.98	72.28	75.90	75.85	81.29	74.74	74.78	78.70	77.83	70.21	69.47	81.61	73.55	71.09
**Antagonist**	67.20	84.15	61.28	64.21	72.97	79.18	78.55	63.50	82.36	75.35	72.48	70.62	89.06	69.83	73.35
**Antagonist**	66.41	74.51	75.41	88.28	66.45	80.55	82.64	79.22	73.55	79.09	68.32	72.18	73.77	70.24	70.08
**Average**	80.45	92.58	81.33	90.93	77.85	103.82	99.29	98.28	94.43	90.04	88.10	83.07	87.88	80.87	73.98

Table S6.. Vina score results for all the molecular targets studied. A more negative score corresponds to a better affinity.

Vina	1MRU	1O6Y	2FUM	3F61	3F69	3ORI	3ORK	3ORL	3ORM	3ORO	3ORP	3ORT	5U94	6B2P	6I2P
**ATP analog**	-8.1	-8.2	-7.6	-9.1	-8.3	-9.6	-8.9	-9.9	-9.3	-9.4	-9.0	-9.1	-8.0	-6.2	-7.8
**ATP analog**	-7.5	-8.2	-7.9	-9.3	-8.4	-9.8	-9.3	-9.5	-9.1	-9.5	-9.4	-8.8	-8.1	-7.1	-7.9
**Antagonist**	-7.2	-7.1	-7.7	-7.6	-8.4	-7.2	-7.5	-8.1	-7.1	-7.3	-7.2	-7.6	-7.5	-6.6	-7.1
**ATP analog**	.7.0	-8.5	-7.6	-8.4	-7.9	-9.2	-8.7	-9.3	-8.9	-8.6	-8.4	-8.4	-7.9	-6.3	-7.3
**Antagonist**	.8.1	-8.1	-8.9	-8.6	-10.4	-8.7	-8.7	-8.9	-8.0	-9.0	-9.2	-8.7	-8.8	-7.7	-7.0
**Antagonist**	-7.4	-8.1	-7.5	-8.0	-8.0	-9.0	-8.3	-8.0	-8.6	-8.5	-7.8	-8.0	-8.9	-7.4	-7.1
**Antagonist**	-7.9	-7.3	-8.7	-8.4	-8.2	-8.2	-8.0	-8.8	-8.3	-8.5	-8.2	-8.3	-7.9	-8.1	-7.5
**Average**	-7.6	-7.9	-8.0	-8.5	-8.5	-8.8	-8.5	-8.9	-8.5	-8.7	-8.5	-8.4	-8.2	-7.1	-7.4

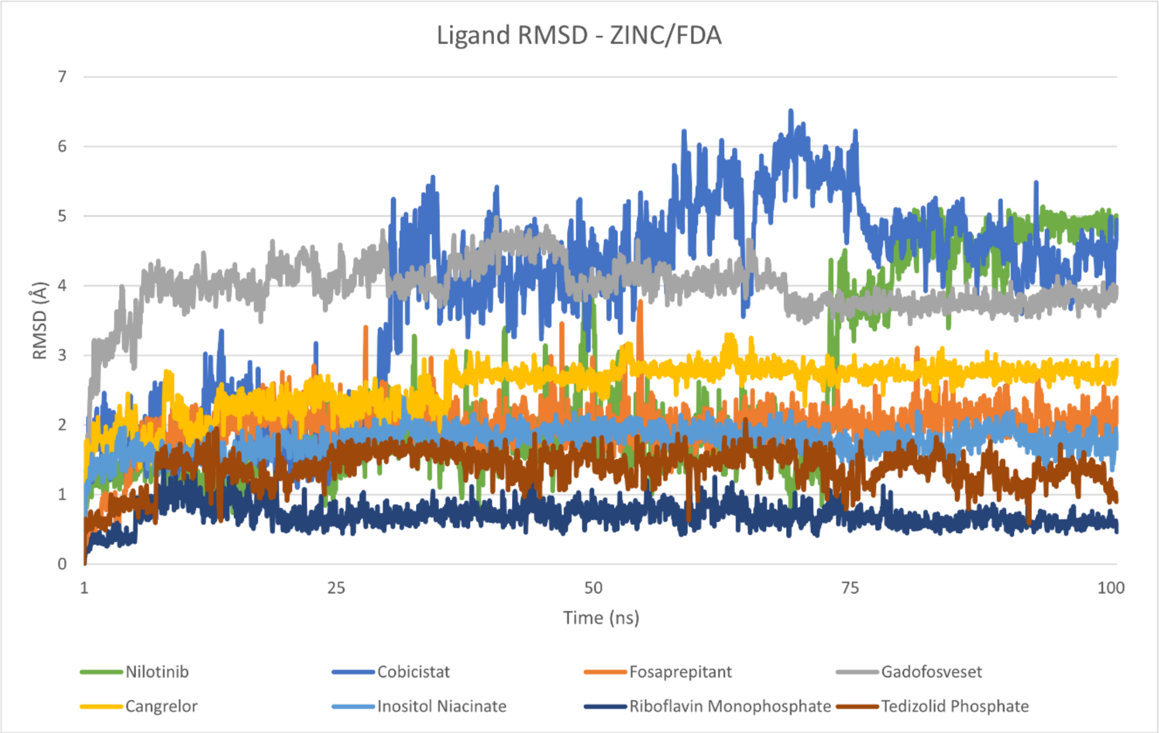

Figure S1 - Root mean square deviation plots for the selected ligands from the ZINC/FDA database.

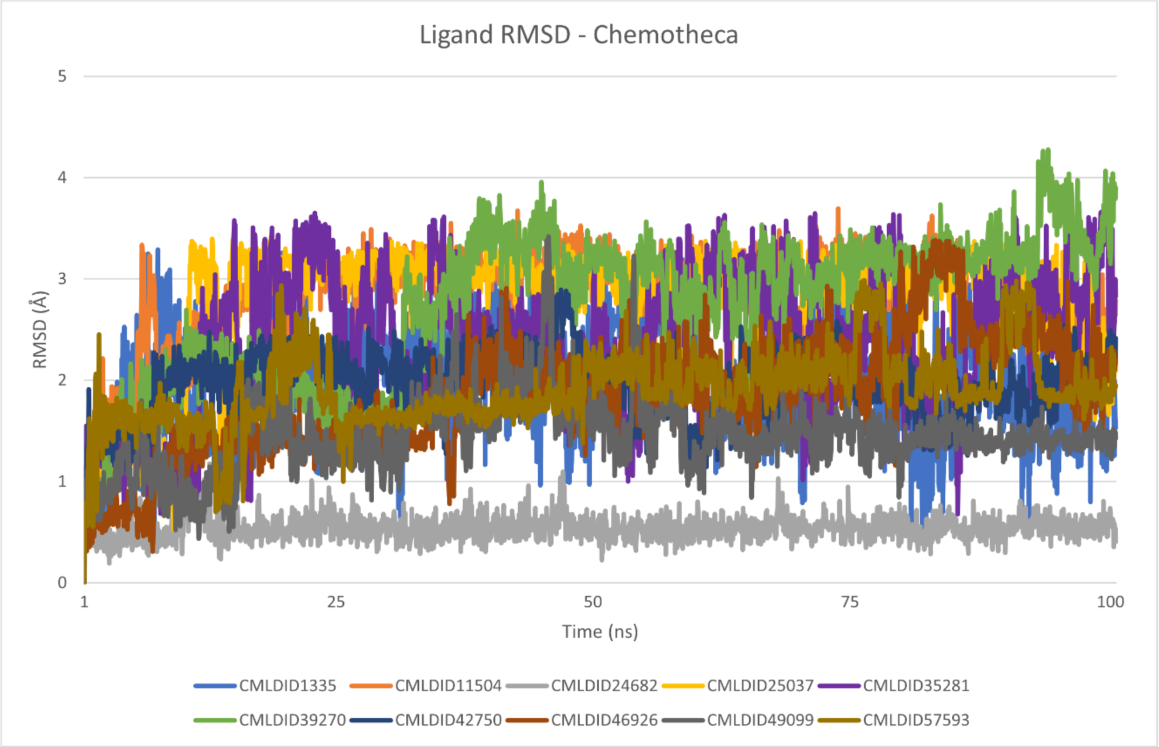

Figure S2 - Root mean square deviation plots for the selected ligands from the Mu.Ta.Lig. Virtual Chemotheca.

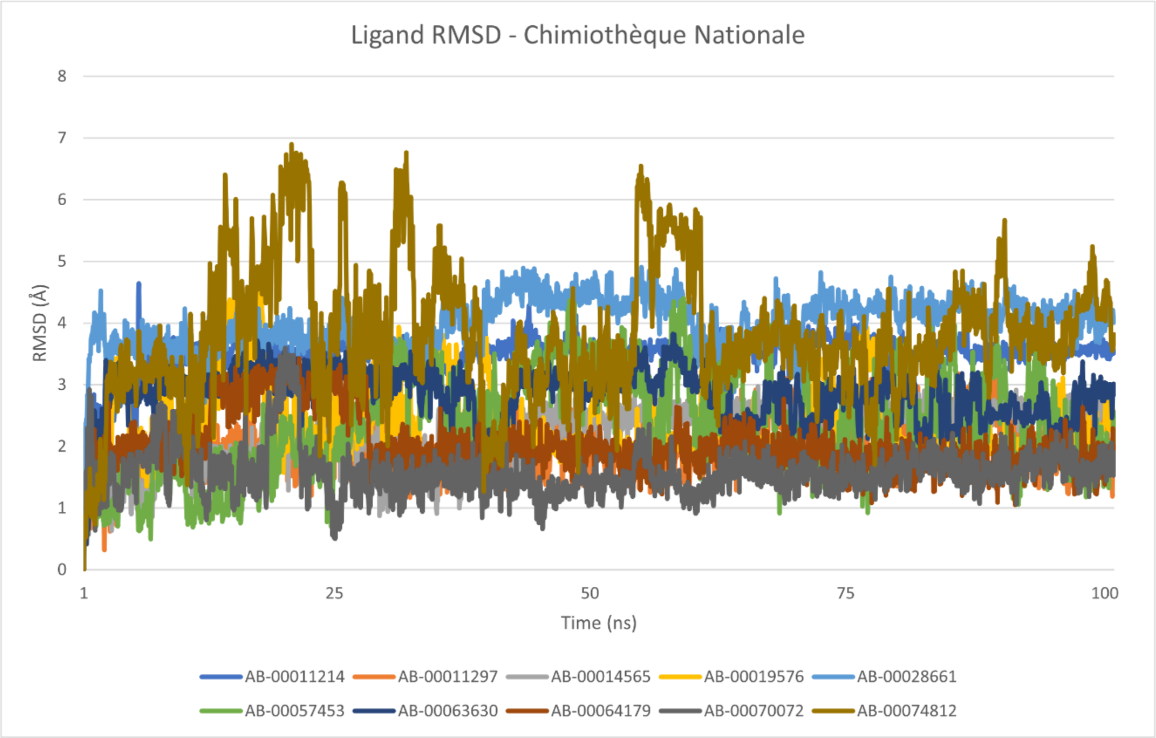

Figure S3 - Root mean square deviation plots for the selected ligands from the Chimiothèque Nationale
